# The Effectiveness of Cancer Pain Management in a Tertiary Hospital Outpatient Pain Clinic in Thailand: A Prospective Observational Study

**DOI:** 10.1155/2021/5599023

**Published:** 2021-07-20

**Authors:** Suratsawadee Wangnamthip, Skaorat Panchoowong, Carolina Donado, Kimberly Lobo, Pimporn Phankhongsap, Pinyo Sriveerachai, Pramote Euasobhon, Pranee Rushatamukayanunt, Sahatsa Mandee, Nantthasorn Zinboonyahgoon, Charles B. Berde

**Affiliations:** ^1^Department of Anesthesiology, Faculty of Medicine Siriraj Hospital, Mahidol University, Bangkok 10700, Thailand; ^2^Department of Anesthesiology, Critical Care and Pain Medicine, Boston Children's Hospital, Boston, MA 02115, USA; ^3^Department of Anaesthesia, Harvard Medical School, Boston, MA 02115, USA; ^4^Siriraj Palliative Care Center, Faculty of Medicine Siriraj Hospital, Mahidol University, Bangkok 10700, Thailand

## Abstract

**Objectives:**

The objective was to examine the effectiveness of the updated approach.

**Methods:**

With IRB approval, outpatients with cancer were enrolled from January to December 2018. Assessments were recorded at baseline and three consecutive visits (BL, FU1, FU2, and FU3), including Numerical Rating Scale (NRS), the Brief Pain Inventory (BPI), the Edmonton Symptom Assessment System (ESAS), side effects, and analgesic use. The primary outcome was a favorable response, defined as an NRS decrease more than 30% or NRS <4. Secondary outcomes included trends over time in BPI, ESAS, side effects, and analgesic use. Pain response predictors at FU3 were analyzed using logistic regression.

**Results:**

Among 150 patients, 72 (48%) completed follow-ups. Of these, 61% achieved a favorable response at FU3. Pain interference diminished at all visits relative to baseline (*p* < 0.05). Median morphine equivalent daily dosage (MEDD) at BL was 20 mg/day, with a statistically significant, but clinically modest increase to 26.4 mg/day at FU3. Radiation therapy during pain care was a predictor of pain responders.

**Conclusion:**

The current Siriraj multidisciplinary approach provided effective relief of pain and stabilization of other cancer-related symptoms. Radiation therapy during pain care can be used to predict pain outcomes. Ongoing improvement domains were identified and considered in the context of cultural, economic, and geographic factors.

## 1. Introduction

Cancer is the second leading cause of death worldwide, with approximately 18.1 million new cases and 9.6 million deaths reported in 2018 [1]. Cancer-related pain is experienced by 50–70% of patients, with a higher prevalence at advanced disease stages (66.4%) [2]. Since the development of WHO's cancer pain guidelines, several studies have reported good relief of symptoms and suffering for a majority of patients [3, 4]. Recent reports suggest that up to 50% of patients still report insufficient pain control [5].

Patients with cancer often present with multiple symptoms and functional decline. Evidence supports multidisciplinary approaches to address symptoms and suffering, including early palliative care referral [6–9].

In 2016, cancer was the second leading cause of death in Thailand [10]. In 2020, there were 190,636 patients with new cancer diagnoses in a population of 69.79 million [11].

Siriraj Hospital is the largest hospital in Thailand and the country's most active cancer treatment center. The Siriraj Pain Clinic has been providing treatment for patients with cancer and noncancer pain since 1989. Two retrospective studies evaluating cancer pain management in outpatients and inpatients conducted in 2014 found that approximately half of the patients with cancer pain seen at the Siriraj pain clinic achieved adequate pain control (30% pain reduction from baseline) [12, 13].

To improve the quality of pain management, the authors and pain clinic team approved and introduced new clinic guidelines for cancer pain management.

A prospective observational study is reported here assessing the impact of pain protocols, nurse-guided analgesic rescue program, selective referral for interventions, early referrals for psychological support and palliative care, and telephone follow-up in addition to standard WHO analgesic guidelines. The overall aim was to assess the effectiveness of this program on measures of pain, other symptoms, and quality of life and to identify persistent barriers to improved treatment.

## 2. Materials and Methods

### 2.1. Study Design

With approval from the Siriraj Institutional Review Board (no. Si 622/2017) and registry with ClinicalTrials.gov (NCT03474406), we conducted a prospective observational study of patients 18 years and older who received new consultation for cancer pain at the Siriraj Pain Clinic from January to December 2018. Written informed consent was obtained from all patients. We excluded patients who had difficulties with listening, reading, and writing Thai and those who were unable to interpret evaluation forms/questionnaires.

All patients enrolled in the study received standard care by pain specialists in the clinic. The WHO analgesic guidelines, selective consideration of pain interventions, and nurse-directed analgesic rescue program were implemented. Additionally, patients received phone calls as reminders to attend follow-up visits. Patients were followed every 2–4 weeks based on physician judgment and pain severity.

### 2.2. Data Collection and Outcome Measures

The primary outcome of this study was the percentage of patients defined as “pain responders” by either 30% reduction in pain intensity rating or pain intensity less than 4 at the third follow-up visit (FU3) [14–16]. Secondary outcomes included pain interference, other symptom scores, side effects, and analgesic prescribing and consumption.

Data were collected at baseline and at three follow-up times (FU1, FU2, and FU3). Patients' baseline demographic data (age, gender, marital status, and education) and clinical characteristics (cancer status, stage, and sites of involvement) were recorded. Pain severity, performance status, pain interference, other symptoms, side effects, pharmacological treatments and dosage, and patient satisfaction were recorded at every visit. We retrospectively reviewed palliative care medical records for additional data related to pain intensity, performance status, opioid consumption, date of first palliative care visit, and dates of death, if applicable. Data were collected by research assistants, nurses, and physicians and entered into Case Report Forms.

### 2.3. Pain Intensity

We used patient-reported seven-day average pain scores (Numerical Rating Scale: NRS) as a primary endpoint, with 0 designating “no pain” and 10 designating “worst possible pain” [17].

### 2.4. Performance Status

Functional impairment was evaluated using the Karnofsky Performance Status (KPS) (100% indicates no functional impairment, no evident disease; 0% indicates death) [18, 19].

### 2.5. Pain Interference and Other Symptoms

We used the validated Brief Pain Inventory (BPI), translated to the Thai language, for measuring pain interference in the last 24 hours, including general activity, mood, walking ability, normal work, relations with other people, sleep, and enjoyment of life. Each category ranges from 0 to 10, where 0 indicates no interference and 10 maximum interference [20]. Patients were regarded as pain interference responders when the total BPI score had 30% reduction or total BPI scores were less than or equal to 28 at FU3 [21, 22].

The common symptoms at the time of assessment in cancer were evaluated using validated Edmonton Symptom Assessment System (ESAS), previously validated and translated to the Thai language, including pain, tiredness, nausea, depression, anxiety, drowsiness, appetite, well-being, and shortness of breath, at the time of assessment, with 0 being no symptoms reported and 10 being the worst possible symptom [23].

### 2.6. Side Effects of Analgesic Drugs

Nausea and vomiting was evaluated using four-point scales [24]. Stool-free interval longer than 72 hours was used to evaluate opioid-induced constipation [25]. Sedation score was used to evaluate opioid-induced sedation [26].

### 2.7. Opioids and Other Analgesics' Use

Morphine equivalent daily dosage (MEDD) [27] was calculated for quantities of opioid prescribed and quantities of opioids taken by patients, including maintenance dosage and total breakthrough doses. Other analgesic drugs recorded included NSAIDs, acetaminophen, anticonvulsants, and antidepressants.

### 2.8. Satisfaction

A three-point scale of patients' satisfaction was used to assess patients' attitudes toward the services of pain clinic as 0, designating satisfied; 1, designating neutral; and 2, designating dissatisfied with services.

### 2.9. Study Size Calculation

In a previous validation study of application of the WHO analgesic ladder, 76% of patients had achieved a good response [3]. Authors expected that average pain intensity should reduce at least 30% [14, 15] at third follow-up (FU3) by 80% of proportion (0.8), 95% confidence level, and 7% allowable error (*d* = 0.7); therefore, the calculated sample size was 126 [28]. Assuming a 20% dropout rate, the total sample size was 150 patients.

### 2.10. Statistical Analysis

Analyses were performed using SPSS statistical package 21.0 (SPSS, Inc., Chicago, IL). Categorical data are reported as numbers and percentages, and continuous data are reported as median (IQR). Repeated measures designs used Friedman tests for continuous data and Cochran's *Q* for dichotomous data, with post hoc comparisons at each time point using Wilcoxon matched-pairs signed rank tests for continuous variables, McNemar tests for dichotomous variables, and marginal homogeneity tests for a categorical variable with 3 or more values. Comparison of continuous variables between two independent populations was assessed using the Mann–Whitney test. Comparison between more than two independent populations was assessed using the Kruskal–Wallis test, with post hoc Dunn–Bonferroni tests between groups. Variables with *p* value <0.1 in the bivariable analyses were considered for entry in subsequent multivariable analyses. A significance level of 0.02 was chosen to account for repeated comparisons across 3 time points.

## 3. Results

### 3.1. Patient Characteristics and Baseline Demographics Data

**Figure 1:**
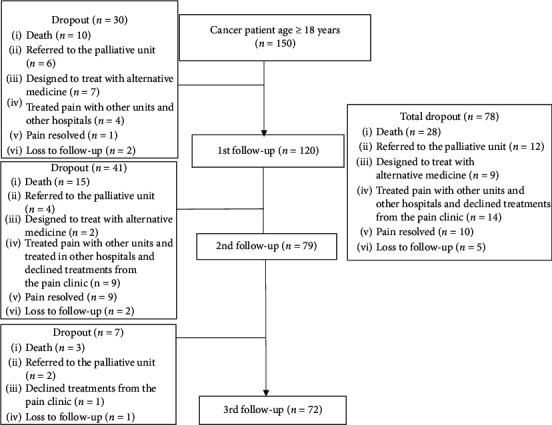
Flow diagram.

A total of 150 newly referred patients with pain due to cancer were enrolled in the study. Of these, 72 patients completed three follow-ups. The remaining 78 patients did not complete three follow-up visits primarily because of death (28 patients; 36%), referral to the palliative care unit (12 patients; 15%), or seeking ongoing care locally (14 patients; 18%) (Figure 1). The median days between follow-ups range from 21.0 to 28.0 [BL-FU1: 21.0 (range = 3.0–74.0), FU1-FU2: 28.0 (range = 5.0–94.0), and FU2-FU3: 28.0 (range = 5.0–91.0)].

A summary of baseline demographics is shown in Table 1. The median age was 58.0 (IQR = 50.0–66.0). Of these, 70% of patients were married, and 62.7% ended their education at a high school level. Most cancers were in advanced stages (80.7%). The common cancer sites were gastrointestinal (26.7%), head and neck (26.7%), and gynecological (14.7%). At baseline, median NRS on seven-day recall was 5.0 (IQR = 4.0–6.0) and median KPS was 70.0 (IQR = 60.0–80.0). The majority of patients were prescribed opioids (85.3%), and the MEDD of opioid taken was 20.0 mg (IQR = 10.0–31.0) before pain clinic referral. Thirty-two percent of patients had a history of radiation therapy before study enrollment. The median of months from diagnosis to the first visit at pain clinic was 9.0 (IQR = 2.0–28.0).

Demographic and clinical features at the initial pain clinic visit were compared between those who subsequently completed all follow-ups in the clinic, those who died during that time period, and those who were referred to palliative care. When compared to those who complete all follow-ups, there was a significantly higher proportion of females referred to palliative care (75.0% vs. 37.5%; *p* = 0.045). Patients referred to palliative care had higher median initial NRS (7.5, IQR = 6.0–8.0) than patients who died during the study period (5.0, IQR = 4.5–6.0; *p*=0.014) and those who completed all follow-ups (5.0, IQR = 4.0–6.0; *p*=0.004). Similarly, patients referred to palliative care had lower initial performance status (50.0, IQR = 40.0–70.0) than those who completed follow-up (70.0, IQR = 60.0–80.0; *p* < 0.001) and those who died (60.0, IQR = 50.0–75.0; *p*=0.003) during the study period.

### 3.2. Pain Scores and KPS

**Figure 2:**
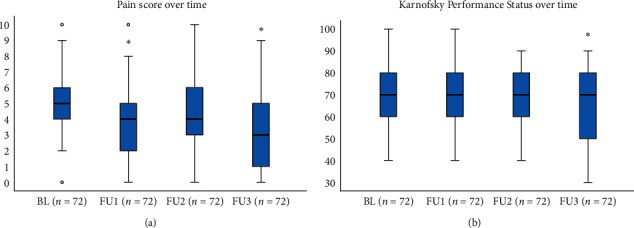
(a) Average pain intensity (7 days recall) at each time point. (b) Karnofsky Performance Status at each time point for patients with complete data. ^∗^*p* value <0.02 using Wilcoxon signed-rank test when comparing each time point to baseline.

The percentage of pain responders at each time point from FU1 to FU3 was 50.0%, 44.3%, and 61.1%, respectively. As shown in Figure 2(a), median of 7 days recall NRS decreased over time from baseline 5.0 (IQR = 4.0–6.0), FU1 4.0 (IQR = 2.0–5.0), FU2 4.0 (IQR = 3.0–6.0), and FU3 3.0 (IQR = 1.0–5.0), respectively (Friedman's test *p* < 0.001). Median KPS functional scores showed a statistically significant but clinically minor decrease over time (Friedman's test *p* < 0.001).

### 3.3. Pain Responders vs. Nonresponders at FU3

In bivariate analyses in baseline demographic data (Table 2), pain responders were more likely to be married (*p*=0.034) and to have received radiation therapy (*p*=0.002) compared to nonresponders. There were no differences in other baselines demographic data, analgesics, and other medication between pain responders and nonresponders.

### 3.4. Pain Interference and Other Symptoms

Table 3 shows the pain interference and common symptom rating by BPI and ESAS. There were significant reductions in overall BPI scores, as well as subscores for relations with others, sleep and enjoyment of life, and no significant changes in walking ability and normal work, from BL to FU3. Median ESAS overall symptom scores decreased over time (Friedman's test, *p*=0.011). The only individual ESAS symptom that decreased to a degree that was statistically significant, but not clinically significant, was the pain intensity (Friedman's test, *p* < 0.001). There were no differences in baseline pain interferences, and other symptoms in pain responders vs. nonresponders (Table 4). Among those with completed FU3, 38 patients met criteria as pain interference responders. In bivariate analysis of baseline demographic factors associated with pain interference responders at FU3, responders had a higher percentage of being married (31 (81.6%) vs. 19 (55.9%), *p*=0.023) and of education not extending past high school (29 (76.3%) vs. 18 (52.9%), *p*=0.049), and there were no differences in other baseline variables such as baseline opioid and medication usage, other symptoms, and side effects; however, none of these predictors remained significant after performing multivariable logistic regression.

### 3.5. Analgesics and Other Medications

Table 5 shows the percentage of patients receiving different analgesic classes, laxatives, and antiemetics. The median dose of opioid prescribed, expressed in MEDD prescribed, did not significantly increase throughout the study (Friedman's test, *p*=0.392). However, opioid consumption, expressed in MEDD taken, did significantly increase over time (Friedman's test, *p*=0.011). The small but significant increase in total daily MEDD taken was seen from FU1 onwards. The median (IQR) opioid ratio percentage [(opioid taken/opioid prescribed)^*∗*^100] was 47.9 (IQR = 29.4–66.7), 49.3 (IQR = 30.7–76.2), 54.2 (IQR = 41.2–93.80), and 52.8 (IQR = 35.7–100) from BL to FU3. The percentage of patients receiving acetaminophen, anticonvulsants, antidepressants, and laxatives also increased significantly at all follow-up time points compared to baseline.

### 3.6. Pain Interventions

There were 6 (4%) patients who underwent pain interventions through the Pain Clinic, including celiac plexus neurolysis, abdominal cutaneous nerve injection, erector spinae plane block, and bilateral pudendal nerve block.

In addition, 29 (19.3%) patients received specialty evaluations from other departments. Sixteen patients were referred to the palliative care unit, four patients were referred to psychiatrists, and the other nine patients were referred to oncologists, general surgeons, hematologists, radiation oncologists, orthopedic surgeons, and internal medicine specialists. 33 patients (22%) received additional radiotherapy during pain care.

### 3.7. Palliative Care Patients

Among the 16 patients referred to palliative care, four (25%) died before the first palliative care visit. Data from the 12 remaining patients' records showed the median of days from the last pain clinic visit to the first palliative care clinic visit was 6.5 days (IQR = 0.0–16.5), the median of days from first palliative care visit to death was 20.0 (IQR = 13.5–34.5), and the median of days from the last pain visit to death was 24.0 (IQR = 15.5–49.0). At the first palliative care visit, the median NRS pain score was 5.0 (IQR = 4.0–8.0), and the median Palliative Performance Status (PPS) was 30% (IQR = 20.0–40.0). The median opioid dosing prescribed at that visit, expressed as MEDD, was 41.0 (IQR = 30.0–66.0). Among the 12 patients referred to palliative care, 7 (58.3%) died at home and all 12 died without cardiopulmonary resuscitation (CPR).

### 3.8. Patients Who Died during the Pain Care

Including the 4 patients who died before the first palliative care visit, 28 patients died during the study period. From the available data on 14 patients, 9 died in hospital (64.3%). One patient received CPR.

Among the 28 patients who died during pain care, 14 (50%) were diagnosed with GI cancer. The median of months from diagnosis to the first pain visit was 8.0 (IQR = 1.0–21.5); however, only seventeen patients had records regarding the death date. The median of months from diagnosis to death was 10.6 (IQR = 7.2–22.3) and the median of days from the last pain visit to death was 16.0 (IQR = 11.0–28.0).

### 3.9. Side Effects

The percentage of moderate-to-severe side effect showed not more than fifteen percent from BL to FU3 (5.6%, 6.9, 8.3%, and 8.3% for sedation; and 13.9%, 11.1%, 8.3% and 12.5% for nausea and vomiting). However, the percentage of moderate-to-severe constipation over time was 12.5% at BL, 15.3% at FU1, and 11.1% at FU2 and reached 23.6% at FU3 (Cochran's *Qp*=0.104).

### 3.10. Predictors of Pain Responders

In bivariate analyses, pain responders were more likely to be married (*p*=0.034) and to have received radiation therapy (*p*=0.002) compared to nonresponders. These factors did not remain significant in multivariable logistic regression models, as shown in Table 6.

Patients were satisfied with cancer pain management in the clinic more than 80% at every visit and reached 88.9% at the third visit.

## 4. Discussion

In this prospective observational study, 61% of outpatients with cancer pain attending the Siriraj Hospital pain clinic were regarded as pain responders as defined above. A previous retrospective study in the Siriraj pain clinic recorded a responder rate of 45%. Based on review of previous experience and in accordance with guidelines, we implemented a multidisciplinary structured approach to management of pain and other symptoms, including guidelines for nurses to provide additional medication for breakthrough pain.

In addition to improvement in pain intensity ratings, patients showed mild improvement or stability in measures of pain interference, and nonpainful symptoms and side effects, and high ratings for satisfaction with treatment. Although a majority of patients showed good responses in terms of pain and other symptoms, there was a remaining subset of patients with persistent moderate-to-severe pain and pain-related disability and interference. Potential interpretations are discussed below.

Response rates to pain management interventions have varied in previous studies of cancer pain treatment in other countries. A retrospective study conducted in Canada showed 53% [29] responders with a pain reduction greater than 30% at the second visit, and 45% of patients responded to cancer pain management at the first visit in a retrospective study conducted in the United States [15].

As in our previous retrospective study, daily opioid consumption expressed as MEDD was comparatively low relative to previously published clinical outcome studies and case series of patients with pain due to cancer in North America, Europe, and East Asia. MEDD taken in this study was approximately 20 mg/day in the beginning and increased to approximately 30 mg/day at the second follow-up. Reported values for median or mean MEDD in case series or outcome studies of patients with advanced cancer in the past decade include 83 mg/day at a center in the United States [30], 60 mg/day at a center in Korea [31], and 121–136 mg/day at a center in Italy [32].

The low value of MEDD relative to published series from other countries did not appear to be due to reluctance among clinic staff to write for larger prescriptions. In particular, during each time interval, patients used less than 50% of the full amount of opioids in their prescriptions. A number of cultural, socioeconomic, and geographic factors may have contributed to this comparatively low value of MEDD taken by patients in our case series.

A study of barriers to cancer pain treatment in Thai patients using the Barriers Questionnaire II found that patients were frequently concerned that greater opioid dosing would generate tolerance, addiction, or inability to monitor changes in one's body [33]. This finding was consistent with a report by Al-Atiyyat et al. indicating that fear of addiction, lack of knowledge regarding opioids, and concern about the side effects are persistent barriers to treatment [34]. A meta-analysis by Chen et al. found that Asian patients had significant higher barrier scores than Western patients due to cultural differences [35].

Thailand is a country undergoing rapid cultural and economic change. Economic development and impact of contact with outside cultures and values is much more evident in Bangkok and is proceeding much more slowly in rural areas and the far north of the country. Buddhism remains the religion of over 90% of people in Thailand. Mindfulness and mental clarity are essential values among many Buddhists and may account for a reluctance to tolerate sedation or mental clouding from opioids [36].

Traditional medicine, including herbs, Thai massage [37], and other modalities, is widely used in Thailand, particularly in rural areas. Nine patients in our case series chose treatment with traditional Thai healers during the course of the study period.

### 4.1. Multidisciplinary Approach

Palliative care for patients with advanced cancer involves symptom management, as well as support around communication, goals, and wishes about end-of-life care, and addressing existential spiritual suffering [9]. Ten percent of the patients in this study had been referred to palliative care. Interestingly, the majority of patients referred for palliative care were women with gynecological cancer. They had higher pain intensity, lower functional status, and higher depression scores than those in completed follow-up patients and patients who died during the pain clinic care. The criteria for palliative care referral in our institute are advanced stage of cancer with functional status KPS less than 50% or specific symptom management. However, there still was a considerable number of patients who died during pain service care without referral to palliative care, and those patients had short survival time; the median time from diagnosis to death was 10.6 months (IQR = 7.2–22.3) and the median time to death from the last pain visit was 16.0 days (IQR = 11.0–28.0), which would be an area for future improvement in our institute. It is plausible that some of the patients who died in hospital might have found support for dying at home with earlier referral for palliative care. Although the American Society of Clinical Oncology (ASCO) recommended that early palliative care service should be implemented in addition to standard care [8], there are still barriers to apply this recommendation due to limited resources and reluctance among some clinicians and/or some patients and families to engage in challenging conversations [38].

### 4.2. Early Pain Intervention

Although the authors implemented access to evidence-based interventional approaches for severe cancer pain [39–41], only four percent of patients underwent pain interventions. Some interventional approaches (e.g., celiac plexus blockade) are effective when pain generators arise from relatively localized tumor burden. In contrast, the majority of our patients came to the clinic at an advanced stage of cancer with multiple pain locations and widespread tumor burden. Other interventional approaches can address more widespread pain, such as implanted intrathecal pumps for administration of opioids and local anesthetics [42]; however, at present these are not covered services in the Thai healthcare system and would require expenses beyond the means of almost all patients.

### 4.3. Predictors of Pain Responders

In our bivariate and multivariate analyses, better responses to pain treatment were associated with palliative radiation therapy during pain care [43–45]. In previous studies, more favorable responses to cancer pain treatment have been associated with being male [29, 46], high socioeconomic status [47], low scores on measures of psychological distress [13], and being married. Factors associated with less favorable response included being female [48], neuropathic pain, and high baseline pain intensity [49].

### 4.4. Opportunities for Improvement

Thailand is regarded by the World Bank as a low-middle income country (LMIC) [50]. Patients from LMICs more often present with an advanced stage of cancer and have a higher mortality rate [51]. Advanced stage of disease often is associated with high pain severity and challenging pain control [47]. Moreover, the limited resources of taking care of advanced-stage cancer with a multidisciplinary approach and early intervention in this setting are still challenging. For many of the patients in this series, there were barriers to follow-up care related to travel distance from towns around the country to Bangkok and limited Internet or mobile phone access. Internet and mobile phone access are expanding greatly in Thailand at present.

In many countries, the COVID-19 pandemic has led to a dramatic acceleration in the effective use of telehealth services. It seems likely that in the future, there could be a greatly expanded role for telehealth in Thailand as a means for providing continuity of care and improving cancer pain management and palliative care services both in the larger cities and throughout the country.

To improve cancer pain management in Thailand, future research may focus on understanding potential barriers associated with comparatively low opioid consumption, use of telehealth for providing ongoing care at a distance from tertiary centers, physicians', patients', and healthcare system perspectives, and consideration of cultural factors in a country undergoing rapid change. In view of the advanced state of disease and short survival of many of the patients in this series, efforts should focus on earlier pain and palliative care referral and better integration of pain and palliative care programs.

### 4.5. Limitation in the Study

This study is a single-site study in a university hospital and tertiary referral center in Bangkok. Patient characteristics and care patterns may not generalize to care throughout the country. Many patients were unable to return to Bangkok for follow-up and returned to local healthcare systems or traditional healers. This led to incomplete information for many patients on their progression of symptoms, responses to pain treatment, and patterns of end-of-life care.

## Figures and Tables

**Table 1:** Baseline demographic data.

	*n* = 150
Age (years), median (IQR)	58.0 (50.0–66.0)
Gender, *n* (%)	
Male	85 (56.7)
Female	65 (43.3)
BMI, median (IQR)	20.7 (18.4–23.3)
Status, *n* (%)	
Married	105 (70.0)
Single/divorced/widowed	45 (30.0)
Living with, *n* (%)	
Family/sibling	143 (95.3)
Alone	7 (4.7)
Education, *n* (%)	
High school and below	94 (62.7)
Higher than high school	56 (37.3)
Pain score (NRS), median (IQR)	5.0 (4.0–6.0)
Karnofsky performance status (%), median (IQR)	70.0 (60.0–80.0)
Cancer status, *n* (%)	
Local	29 (19.3)
Advanced	121 (80.7)
Primary tumor, *n* (%)	
Gastrointestinal	40 (26.7)
Bronchus and lung	19 (12.7)
Breast	9 (6.0)
Head and neck	40 (26.7)
Hematological	4 (2.7)
Gynecological	22 (14.7)
Urological	12 (8.0)
Musculoskeletal	4 (2.7)
Pain medications taken, *n* (%)	
Opioids	128 (85.3)
Acetaminophen	70 (46.7)
NSAIDs	14 (9.3)
Anticonvulsants	57 (38.0)
Antidepressants	21 (14.0)
Laxatives	53 (35.3)
Antiemetics	10 (6.7)
MEDD prescription (mg/day), median (IQR)	50.0 (15.0–82.0)
MEDD taken (mg/day), median (IQR)	20.0 (10.0–31.0)
History of radiation before study, *n* (%)	48 (32.0)
Duration (days) from diagnosis, median (IQR)	296.0 (76.0–855.0)

BMI: Body Mass Index; NRS: Numerical Rating Scale; NSAIDs: Nonsteroidal Anti-Inflammatory Drugs; MEDD: Morphine Equivalent Diary Dosage.

**Table 2:** Demographic data difference between pain responders and nonresponders at FU3.

	Pain responders, *n* = 44	Nonresponders, *n* = 28	*p* value
Age (years), median (IQR)	56.5 (47.0–65.5)	58.0 (49.0–65.5)	0.871
Gender, *n* (%)			0.225
Male	30 (68.2)	15 (53.6)	
Female	14 (31.8)	13 (46.4)	
BMI, median (IQR)	21.5 (17.9–24.2)	21.5 (17.6–23.6)	0.917
Status, *n* (%)			0.034^*∗*^
Married	35 (79.5)	15 (53.6)	
Single/divorced/widowed	9 (20.5)	13 (46.4)	
Living with, *n* (%)			1.000
Family/sibling	42 (95.5)	27 (96.4)	
Alone	2 (4.5)	1 (3.6)	
Education, *n* (%)			0.312
High school and below	31 (70.5)	16 (57.1)	
Higher than high school	13 (29.5)	12 (42.9)	
Pain score (NRS), median (IQR)	5.0 (4.0–7.0)	5.0 (4.0–6.0)	0.617
KPS (%), median (IQR)	70.0 (60.0–80.0)	75.0 (60.0–80.0)	0.807
Cancer status, *n* (%)			0.066
Local	11 (25.0)	2 (7.1)	
Advanced	33 (75.0)	26 (92.9)	
Primary tumor, *n* (%)			0.821
Gastrointestinal	8 (18.2)	7 (25.0)	
Bronchus and lung	8 (18.2)	4 (14.3)	
Breast	2 (4.5)	1 (3.6)	
Head and neck	17 (38.6)	7 (25.0)	
Hematological	1 (2.3)	0 (0)	
Gynecological	4 (9.1)	5 (17.9)	
Urological	3 (6.8)	3 (10.7)	
Musculoskeletal	1 (2.3)	1 (3.6)	
Pain medications taken, *n* (%)			
Opioids	39 (88.6)	24 (85.7)	0.728
Acetaminophen	20 (45.5)	13 (46.4)	1.000
NSAIDs	6 (13.6)	2 (7.1)	0.471
Anticonvulsants	16 (36.4)	16 (57.1)	0.095
Antidepressants	5 (11.4)	5 (17.9)	0.496
Laxatives	18 (40.9)	9 (32.1)	0.618
Antiemetics	4 (9.1)	3 (10.7)	1.000
MEDD prescription, median (IQR)	50.0 (16.0–80.0)	68.0 (12.0–91.0)	0.768
MEDD taken, median (IQR)	20.0 (10.0–30.0)	24.5 (10.0–39.0)	0.625
History of radiation therapy before study	18 (40.9)	12 (42.9)	1.000
History of radiation during pain care	18 (40.9)	2 (7.1)	0.002^*∗*^

BMI: Body Mass Index; NRS: Numerical Rating Scale; NSAIDs: Nonsteroidal Anti-Inflammatory Drugs; MEDD: Morphine Equivalent Diary Dosage. ^*∗*^*p* < 0.05 indicates statistically significant difference using Mann–Whitney *U* test and Chi-square test.

**Table 3:** Pain interference rating by BPI and other symptoms' rating per ESAS.

	BL (*n* = 72)	FU1 (*n* = 72)	FU2 (*n* = 72)	FU3 (*n* = 72)	*p* value (Friedman test)
BPI, pain interference, median (IQR)					
General activity	6.0 (3.5–8.0)	4.0 (0.0–7.0)^*∗*^	4.5 (1.0–7.0)^*∗*^	5.0 (0.0–7 .0)^*∗*^	0.004
Mood	5.5 (2.0–8.0)	5.0 (1.0–7.0)	3.0 (0.0–6.5)^*∗*^	3.0 (0.0–6.5)^*∗*^	0.012
Walking ability	5.0 (2.5–8.0)	5.0 (0.0–8.0)	5.0 (0.0–8.5)	5.0 (0.0–8.0)	0.191
Normal work	6.0 (3.0–9.0)	4.5 (0.0–9.0)	5.0 (0.5–9.0)	5.0 (0.0–8.0)	0.407
Relations with others	4.5 (1.0–7.0)	2.0 (0.0–5.0)^*∗*^	3.0 (0.0–5.0)	1.5 (0.0–4.5)^*∗*^	0.002
Sleep	7.0 (4.0–8.0)	5.0 (0.0–7.5)^*∗*^	4.0 (0.0–7.0)^*∗*^	2.5 (0.0–6.0)^*∗*^	<0.001
Enjoyment of life	5.0 (2.0–8.0)	5.0 (2.0–8.0)	4.5 (0.5–7.5)	3.5 (0.0–6.0)^*∗*^	0.017
BPI total score	40.0 (26.0–52.0)	29.5 (15.0–44.0)^*∗*^	30.0 (14.0–46.0)^*∗*^	26.0 (8.5–40.5)^*∗*^	<0.001
ESAS, symptoms, median (IQR)					
Pain	4.0 (2.5–6.0)	4.0 (2.0–5.5)	3.0 (1.0–6.0)^*∗*^	3.0 (1.0–5.5)^*∗*^	<0.001
Fatigue	5.0 (2.0–6.0)	5.0 (2.0–6.0)	4.0 (0.0–6.0)	4.5 (2.0–6.0)	0.173
Nausea	0.0 (0.0–3.0)	0.0 (0.0–2.0)	0.0 (0.0–1.0)	0.0 (0.0–1.5)	0.152
Depression	0.0 (0.0–3.0)	0.0 (0.0–2.0)	0.0 (0.0–2.5)	0.0 (0.0–2.5)	0.712
Anxiety	3.0 (0.0–5.0)	2.0 (0.0–3.0)	1.0 (0.0–5.0)	1.0 (0.0–5.0)	0.204
Drowsiness	3.0 (0.5–5.0)	2.0 (0.0–5.0)	2.0 (0.5–4.0)	2.0 (0.0–5.5)	0.806
Appetite	4.0 (0.0–7.0)	3.0 (0.05.0)	3.0 (0.0–5.0)	2.5 (0.0–5.0)	0.094
Well-being	4.5 (0.0–6.5)	4.0 (0.5–5.0)	2.0 (0.0–5.0)	3.0 (0.0–6.0)	0.729
Shortness of breath	2.0 (0.0–5.0)	2.0 (0.0–5.0)	1.0 (0.0–4.5)	1.0 (0.0–5.0)	0.438
ESAS total score	27.5 (17.0–43.0)	23.0 (12.0–36.0)	17.5 (10.5–32.0)^*∗*^	23.5 (10.5–36.5)	0.011

^*∗*^ *p* < 0.02 at follow-up compared to baseline using Wilcoxon signed-rank test. FU: follow-up; BPI: Brief Pain Inventory; ESAS: Edmonton Symptom Assessment System.

**Table 4:** Pain interferences and other symptoms' difference between pain responders and nonresponders at FU3.

	Pain responders (*n* = 44)	Nonresponders (*n* = 28)	*p* value
BPI, pain interference, median (IQR)			
General activity	6.0 (4.0–9.5)	6.0 (2.5–8.0)	0.276
Mood	6.0 (2.5–8.0)	5.0 (2.0–7.5)	0.496
Walking ability	5.0 (2.5–8.0)	5.5 (2.5–8.0)	0.780
Normal work	6.0 (2.5–10.0)	5.0 (3.0–8.0)	0.548
Relations with others	4.5 (0.5–7.0)	4.5 (1.0–6.5)	0.986
Sleep	7.0 (4.5–8.5)	7.0 (4.0–8.0)	0.954
Enjoyment of life	6.0 (2.0–9.0)	5.0 (2.0–8.0)	0.481
BPI total score	40.5 (26.0–52.5)	39.5 (25.5–46.5)	0.591
ESAS, symptoms, median (IQR)			
Pain	4.0 (2.5–5.0)	5.0 (2.5–7.0)	0.085
Fatigue	4.5 (2.0–6.5)	5.0 (2.0–6.0)	1.000
Nausea	0.0 (0.0–2.0)	0.5 (0.0–3.0)	0.489
Depression	0.0 (0.0–2.5)	0.0 (0.0–3.5)	0.732
Anxiety	2.5 (0.0–5.0)	3.0 (1.0–5.0)	0.262
Drowsiness	2.5 (0.5–5.0)	3.0 (0.5–5.0)	0.806
Appetite	3.5 (0.0–7.5)	4.0 (1.0–5.5)	0.774
Well-being	3.5 (0.0–7.0)	5.0 (0.5–5.5)	0.986
Shortness of breath	2.0 (0.0–5.0)	3.0 (0.0–5.5)	0.569
ESAS total score	26.5 (15.5–42.0)	30.0 (18.0–44.0)	0.595

^*∗*^ *p* < 0.05 at follow-up compared to baseline using Mann–Whitney *U* test. FU: follow-up; BPI: Brief Pain Inventory; ESAS: Edmonton Symptom Assessment System.

**Table 5:** Opioid prescription pain medication taken in completed follow-up patients.

	BL (*n* = 72)	FU1 (*n* = 72)	FU2 (*n* = 72)	FU3 (*n* = 72)	*p* value (Cochran's *Q*, Friedman's test)
Opioid prescribed	68 (94.4)	69 (95.8)	67 (93.1)	65 (90.3)	0.779
MEDD prescribed, mg/day, median (IQR)	57.0 (15.0–86.0)	68.0 (11.2–100.0)	68.0 (12.5–92.0)	63.0 (10.0–92.0)	0.392
Opioid taken	63 (87.5)	69 (95.8)	67 (93.1)	65 (90.3)	0.112
MEDD taken, mg/day, median (IQR)	20.0 (10.0–36.5)	24.0 (10.0–38.0)^*∗*^	28.0 (11.3–49.0)^*∗*^	26.4 (10.0–50.0)^*∗*^	0.011^*∗*^
Nonopioid medication prescribed					
Acetaminophen	33 (45.8)	48 (66.7)^*∗*^	47 (65.3)^*∗*^	45 (62.5)	0.003
NSAIDs	8 (11.1)	11 (15.3)	9 (12.5)	6 (8.3)	0.409
Anticonvulsants	32 (44.4)	62 (86.1)^*∗*^	62 (86.1)^*∗*^	59 (81.9)^*∗*^	<0.001^*∗*^
Antidepressants	10 (13.9)	21 (29.2)^*∗*^	28 (38.9)^*∗*^	25 (34.7)^*∗*^	<0.001^*∗*^
Laxatives	27 (37.5)	51 (70.8)^*∗*^	47 (65.3)^*∗*^	47 (65.3)^*∗*^	<0.001^*∗*^
Antiemetics	7 (9.7)	4 (5.6)	8 (11.1)	5 (6.9)	0.414

Except for the row with MEDD, data are presented as number (percentage). ^*∗*^*p* < 0.02 at follow-up compared to baseline using McNemar test and Wilcoxon signed-rank test. MEDD: Morphine Equivalent Daily Dosage.

**Table 6:** Multivariable logistic regression results for predicting pain responders at FU3.

Predictor variables	Multivariate analyses		
	OR	95% CI	*p* value
Younger age	0.987	0.9–1.0	0.600
Male gender	1.474	0.4–4.8	0.523
Marriage	3.220	0.9–11.2	0.067
Local cancer status	3.480	0.6–19.2	0.152
Lower baseline ESAS pain	0.802	0.6–1.0	0.076
Radiation during pain care	6.715	1.3–34.4	0.022

ESAS: Edmonton Symptom Assessment System.

## Data Availability

The SPSS data used to support the findings of this study are available from Dr. Wangnamthip (e-mail: suratsawadee.wan@mahidol.ac.th) upon request.

## References

[1] Bray F., Ferlay J., Soerjomataram I., Siegel R. L., Torre L. A., Jemal A. (2018). Global cancer statistics 2018: GLOBOCAN estimates of incidence and mortality worldwide for 36 cancers in 185 countries. *CA: A Cancer Journal for Clinicians*.

[2] Van Den Beuken-Van Everdingen M. H. J., Hochstenbach L. M. J., Joosten E. A. J., Tjan-Heijnen V. C. G., Janssen D. J. A. (2016). Update on prevalence of pain in patients with cancer: systematic review and meta-analysis. *Journal of Pain and Symptom Management*.

[3] Zech D. F. J., Grond S., Lynch J., Hertel D., Lehmann K. A. (1995). Validation of World Health Organization Guidelines for cancer pain relief: a 10-year prospective study. *Pain*.

[4] Carlson C. (2016). Effectiveness of the World Health Organization cancer pain relief guidelines: an integrative review. *Journal of Pain Research*.

[5] Neufeld N. J., Elnahal S. M., Alvarez R. H. (2017). Cancer pain: a review of epidemiology, clinical quality and value impact. *Future Oncology*.

[6] Banning A., Sjogren P., Henriksen H. (1991). Treatment outcome in a multidisciplinary cancer pain clinic. *Pain*.

[7] Chen J., Lu X.-Y., Wang W.-J. (2014). Impact of a clinical pharmacist-led guidance team on cancer pain therapy in China: a prospective multicenter cohort study. *Journal of Pain and Symptom Management*.

[8] Ferrell B. R., Temel J. S., Temin S. (2017). Integration of palliative care into standard Oncology care: American society of clinical Oncology clinical practice guideline update. *Journal of Clinical Oncology*.

[9] Sayed D. (2013). The interdisciplinary management of cancer pain. *Techniques in Regional Anesthesia and Pain Management*.

[10] WHO (2018). *Noncommunicable Diseases Country Profiles 2018*.

[11] World Health Organization (2020). *Thailand Population Fact Sheets: Globocan 2020*.

[12] Euasobhon P., Wangnamthip S., Payomyam C. (2019). Cancer pain management: is it still problematic?. *Siriraj Medical Journal*.

[13] Wangnamthip S., Euasobhon P., Siriussawakul A., Jirachaipitak S., Laurujisawat J., Vimolwattanasarn K. (2016). Effective pain management for inpatients at Siriraj hospital: a retrospective study. *Journal of the Medical Association of Thailand*.

[14] Farrar J. T., Young J. P., LaMoreaux L., Werth J. L., Poole M. R. (2001). Clinical importance of changes in chronic pain intensity measured on an 11-point numerical pain rating scale. *Pain*.

[15] Yennurajalingam S., Kang J. H., Hui D., Kang D.-H., Kim S. H., Bruera E. (2012). Clinical response to an outpatient palliative care consultation in patients with advanced cancer and cancer pain. *Journal of Pain and Symptom Management*.

[16] Paul S. M., Zelman D. C., Smith M., Miaskowski C. (2005). Categorizing the severity of cancer pain: further exploration of the establishment of cutpoints. *Pain*.

[17] Hawker G. A., Mian S., Kendzerska T., French M. (2011). Measures of adult pain: visual analog scale for pain (VAS pain), numeric rating scale for pain (NRS pain), McGill pain questionnaire (MPQ), short-form McGill pain questionnaire (SF-mpq), chronic pain grade scale (CPGS), short form-36 bodily pain scale (SF-36 BPS), and measure of intermittent and constant osteoarthritis pain (ICOAP). *Arthritis Care Res (Hoboken)*.

[18] Crooks V., Waller S., Smith T., Hahn T. J. (1991). The use of the Karnofsky Performance Scale in determining outcomes and risk in geriatric outpatients. *Journal of Gerontology*.

[19] Péus D., Newcomb N., Hofer S. (2013). Appraisal of the karnofsky performance status and proposal of a simple algorithmic system for its evaluation. *BMC Medical Informatics and Decision Making*.

[20] Chaudakshetrin P. (2009). Validation of the Thai version of brief pain inventory (BPI-T) in cancer patients. *Journal of the Medical Association of Thailand*.

[21] Dworkin R. H., Turk D. C., Wyrwich K. W. (2008). Interpreting the clinical importance of treatment outcomes in chronic pain clinical trials: IMMPACT recommendations. *The Journal of Pain*.

[22] Serlin R. C., Mendoza T. R., Nakamura Y., Edwards K. R., Cleeland C. S. (1995). When is cancer pain mild, moderate or severe? Grading pain severity by its interference with function. *Pain*.

[23] Chinda M., Jaturapatporn D., Kirshen A. J., Udomsubpayakul U. (2011). Reliability and validity of a Thai version of the edmonton symptom assessment scale (ESAS-Thai). *Journal of Pain and Symptom Management*.

[24] Watcha M. F., White P. F. (1992). Postoperative nausea and vomiting. *Anesthesiology*.

[25] Wirz S., Klaschik E. (2005). Management of constipation in palliative care patients undergoing opioid therapy: is polyethylene glycol an option?. *American Journal of Hospice and Palliative Medicine*.

[26] Pasero C. (2009). Assessment of sedation during opioid administration for pain management. *Journal of Peri Anesthesia Nursing*.

[27] Hoots B. E., Xu L., Kariisa M. (2018). 2018 annual surveillance report of drug-related risks and outcomes—United States. *CDC’s Injury Center*.

[28] Elashoff J., Crede F. (2007). *nQuery Advisor (versión 7.0) [Software de computación]*.

[29] Perez J., Olivier S., Rampakakis E., Borod M., Shir Y. (2016). The McGill university health centre cancer pain clinic: a retrospective analysis of an interdisciplinary approach to cancer pain management. *Pain Research & Management*.

[30] Yennurajalingam S., Lu Z., Reddy S. K. (2019). Patterns of opioid prescription, use, and costs among patients with advanced cancer and inpatient palliative care between 2008 and 2014. *Journal of Oncology Practice*.

[31] Kwon J. H., Oh S. Y., Chisholm G. (2013). Predictors of high score patient-reported barriers to controlling cancer pain: a preliminary report. *Supportive Care in Cancer*.

[32] Mercadante S., Porzio G., Adile C. (2015). Pain intensity as prognostic factor in cancer pain management. *Pain Practice*.

[33] Worakul W., Petpichetchian W., Nilmanat K. (2008). Barriers to pharmacological management of cancer pain: a comparison of beliefs between patients and caregivers. *Thai Journal of Nursing Council*.

[34] Al-Atiyyat N., MSNc R. (2016). Barriers toward using opioids in cancer pain management. *Journal of Nursing and Health Science*.

[35] Chen C. H., Tang S. T., Chen C. H. (2012). Meta-analysis of cultural differences in Western and Asian patient-perceived barriers to managing cancer pain. *Palliative Medicine*.

[36] Smith-Toner M. (2003). How Buddhism influences pain control choices. *Nursing*.

[37] Lumlerdkij N., Tantiwongse J., Booranasubkajorn S. (2018). Understanding cancer and its treatment in Thai traditional medicine: an ethnopharmacological-anthropological investigation. *Journal of Ethnopharmacology*.

[38] Nilmanat K. (2016). Palliative care in Thailand: development and challenges. *Clinical Journal of Oncology Nursing*.

[39] Amr Y. M., Makharita M. Y. (2014). Neurolytic sympathectomy in the management of cancer pain-time effect: a prospective, randomized multicenter study. *Journal of Pain and Symptom Management*.

[40] Dobosz Ł., Stefaniak T., Dobrzycka M. (2016). Invasive treatment of pain associated with pancreatic cancer on different levels of WHO analgesic ladder. *BMC Surgery*.

[41] Smith T. J., Staats P. S., Deer T. (2002). Randomized clinical trial of an implantable drug delivery system compared with comprehensive medical management for refractory cancer pain: impact on pain, drug-related toxicity, and survival. *Journal of Clinical Oncology*.

[42] Stearns L. J., Narang S., Albright R. E. (2019). Assessment of health care utilization and cost of targeted drug delivery and conventional medical management vs conventional medical management alone for patients with cancer-related pain. *JAMA Network Open*.

[43] Chow E., Zeng L., Salvo N., Dennis K., Tsao M., Lutz S. (2012). Update on the systematic review of palliative radiotherapy trials for bone metastases. *Clinical Oncology*.

[44] Macleod N., Price A., O’Rourke N., Fallon M., Laird B. (2014). Radiotherapy for the treatment of pain in malignant pleural mesothelioma: a systematic review. *Lung Cancer*.

[45] Rich S. E., Chow R., Raman S. (2018). Update of the systematic review of palliative radiation therapy fractionation for bone metastases. *Radiotherapy and Oncology*.

[46] Chou P.-L., Fang S.-Y., Sun J.-L., Rau K.-M., Lee B.-O. (2018). Gender difference in cancer patients’ adherence to analgesics and related outcomes of pain management. *Cancer Nursing*.

[47] Wang H.-L., Kroenke K., Wu J., Tu W., Theobald D., Rawl S. M. (2012). Predictors of cancer-related pain improvement over time. *Psychosomatic Medicine*.

[48] Reis-Pina P., Lawlor P. G., Barbosa A. (2017). Adequacy of cancer-related pain management and predictors of undertreatment at referral to a pain clinic. *Journal of Pain Research*.

[49] Arthur J., Tanco K., Park M. (2018). Personalized pain goal as an outcome measure in routine cancer pain assessment. *Journal of Pain and Symptom Management*.

[50] *World Bank Country and Lending Groups*.

[51] Shah S. C., Kayamba V., Peek R. M., Heimburger D. (2019). Cancer control in low- and middle-income countries: is it time to consider screening?. *Journal of Global Oncology*.

